# Organ-Specific Metabolite Profiling of *Mahonia aquifolium* (Pursh) Nutt. Extracts by GC-FID/MS and UHPLC-HRMS/MS with Bioactivity Assessment

**DOI:** 10.1021/acsomega.5c12812

**Published:** 2026-03-04

**Authors:** Kübra Öğüt, Elif Kaya Tilki, Ana M. Troncoso, Temel Özek

**Affiliations:** † 52944 Anadolu University Department of Pharmacognosy, Faculty of Pharmacy, Eskisehir 26470, Türkiye; ‡ Anadolu University, Department of Pharmacology, Eskisehir 26470, Türkiye; § Departamento de Nutrición y Bromatología, Toxicología y Medicina Legal, Facultad de Farmacia, 16778 Universidad de Sevilla, C/P García González No. 2, Sevilla 41012, Spain

## Abstract

*Mahonia aquifolium* (Pursh) Nutt., an ornamental shrub abundant in bioactive metabolites, was investigated using an organ-specific metabolite profiling approach. GC-FID/MS (after silylation) and UHPLC-HRMS/MS were employed to qualitatively and quantitatively characterize the constituents in extracts. The leaf extract exhibited the highest phenolic and flavonoid contents. 3-Hydroxytyrosol and flavanomarein were identified for the first time in specific plant organs. Antioxidant capacity and α-amylase inhibitory activity were evaluated using several *in vitro* assays. Cytotoxicity was evaluated on NIH/3T3 fibroblast cells and PMA-differentiated THP-1 macrophage-like cells. At the tested concentrations, none of the extracts exhibited relevant antiproliferative activity, with IC_50_ values above 400 μg/mL for all samples; only the leaf extract showed a mild, time-dependent reduction in cell viability. These findings indicate limited cytotoxic activity at the applied doses. This study provides the first comprehensive polar metabolite and bioactivity map of four anatomical regions of *M. aquifolium*, highlighting organ-specific chemical diversity and potential applications in functional food and nutraceutical development.

## Graphical abstract

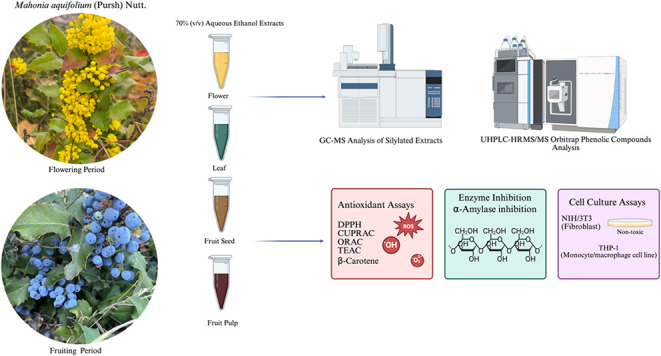

## 1 Introduction

The Berberidaceae family comprises nine genera and approximately 590 species, primarily distributed across South America and the Northern Hemisphere.^1^ Among the species in the genus, *Mahonia aquifolium* (Pursh) Nutt. is a perennial woody shrub notable for its invasive behavior and vibrant, fragrant yellow flowers that typically bloom in April. Its purple fruits, commonly known as Oregon grapes, have contributed to the species’ broad recognition. Native to western North America, *M. aquifolium* has successfully naturalized in various parts of the Americas, Australia, and Europe, where it is appreciated for both decorative and medicinal purposes. *M. aquifolium* is also widely recognized as an ornamental shrub, frequently used in landscaping due to its evergreen foliage, resilience, and decorative flowering habit.^2^ In Türkiye, *Mahonia* species (particularly *M. aquifolium*) are widely cultivated as ornamental plants in parks and gardens, especially in humid regions.^3^ This widespread cultivation underscores the species’ ecological adaptability and ornamental value, which have made it a favored element in landscape architecture. Moreover, its long-standing use in traditional medicine further contributes to its ethnobotanical and pharmacological significance across different cultural regions.^2,4,5^ In traditional Chinese medicine (TCM), various *Mahonia* species, particularly stems and leaves of *Mahonia belai* (Fortune) Carrière and *Mahonia fortune* (Lindl.) Fedde, which are officially recognized in the Chinese Pharmacopoeia under the name “*Mahonia caulis*,” are used to clear internal heat, eliminate dampness, detoxify the body, alleviate pain, promote blood circulation, suppress cough, and reduce inflammation.^5^ *M. aquifolium* has been used in traditional medicine for treating psoriasis, dermatitis, fungal infections, tuberculosis, dysentery, and wounds.^6–9^ Besides its traditional medicinal uses, the fruits of the genus *Mahonia* are frequently designated in the literature as “edible wild berries” and have been widely utilized in the preparation of jams, marmalades, and similar culinary products across different regions.^10,11^

Current global trends in plant science highlight the increasing focus on secondary metabolite research, particularly for their significance in pharmacology, stress resilience, and ecological adaptability. Progress in biotechnology and *in vitro* production systems has underscored the necessity for precise characterization of natural metabolite patterns to inform cultivation and conservation strategies.^12,13^

*Mahonia* species are widely recognized for their richness in phenolic compounds, which play a key role in enhancing their antioxidant capacity. The specific compounds and their concentrations are known to vary depending on the species, plant organ, and stage of development. Phenolic acids, flavonoids, anthocyanins, and tannins have been reported for *Mahonia* species.^3,14^ Furthermore, a variety of secondary metabolites, including sterols, benzoquinones, and lignans, have also been identified in *Mahonia* species.^15^ Phenolic and other polar metabolites are crucial aspects of the nutritional quality, antioxidant capacity, and functional food value of plant-derived products.^16^ Despite increasing interest, no study has systematically compared the polar metabolite composition of multiple anatomical parts of *M. aquifolium* within a single investigation. Additionally, recent studies have demonstrated that species of the genus *Mahonia* possess a wide range of pharmacological activities attributed to their rich phytochemical composition. Also, *Mahonia* species exhibit antioxidant, antimicrobial, and antifungal activities, mainly through alkaloids such as berberine, which impair microbial growth and integrity.^9,17,18^ Antitumor effects have also been reported, supported by evidence of inhibited cancer cell proliferation and modulation of immune cell functions.^19^ Several *Mahonia* species showed immunomodulatory activities by influencing cytokine production and immune regulation.^20^ These bioactivities collectively highlight the therapeutic potential of *Mahonia* species for various health applications.^5,19^

This study aims to comprehensively characterize the phytochemical profiles of polar extracts obtained from the leaves, flowers, fruit pulp, and seeds of *M. aquifolium* and to evaluate their antioxidant and enzyme inhibitory activities. Structural elucidation of the polar constituents was performed using both UHPLC-HRMS/MS and GC-FID/MS analysis following silylation, offering complementary perspectives on the chemical composition of the extracts. These high-resolution analytical approaches are widely applied in contemporary food chemistry to characterize bioactive metabolites and evaluate their potential health-related properties. Recent investigations of *Mahonia* species in the past five years have predominantly concentrated on alkaloid-rich bark, with less emphasis on polar extracts and generally confined to a singular plant part. Although GC–FID/MS and UHPLC-HRMS/MS are widely used analytical platforms, they have not previously been applied in an integrated manner to characterize organ-specific polar metabolites of *M. aquifolium*. This gap underscores the need for a combined analytical approach to fully resolve the polar metabolite diversity of this species. This dual-platform methodology enhances the analytical scope of polar extracts beyond the capabilities of single-method techniques, providing a more comprehensive strategy for metabolite profiling in intricate botanical matrices. By integrating both chemical and biological assessments of polar extracts from distinct plant parts, this study offers a comprehensive and focused perspective on the bioactive potential of *M. aquifolium*, addressing an existing gap in the phytochemical literature.

## 2 Materials and Methods

### 2.1 Chemicals

Methanol, ethanol, formic acid, hydrochloric acid, glacial acetic acid, Folin–Ciocalteu reagent (FCR), dimethyl sulfoxide (DMSO), *N*,*O*-bis­(trimethylsilyl)­trifluoroacetamide (BTSFA, with 1% TMCS) and butylated hydroxyanisole (BHA) were purchased from Sigma-Aldrich. *n*-Hexane and other general-purpose reagents were sourced from Merck (Germany). A homologous series of *n*-alkanes (C_8_–C_40_) for retention index calculations was obtained from Fluka (Buchs, Switzerland). α-Amylase from porcine pancreas (Type VI–B, ≥10 units/mg solid), acarbose (used as a reference α-amylase inhibitor) were obtained from Sigma-Aldrich (St. Louis, MO). Phenolic standards used in qualitative and quantitative analysis were purchased from Merck (Darmstadt, Germany), and included abscisic acid, apigenin, apigenin-7-*O*-glucoside, aromadendrin, 3-hydroxytyrosol, 4-hydroxybenzoic acid, 4-*O*-caffeoylquinic acid, caffeic acid, dihydrocaffeic acid, catechin, chlorogenic acid, *p*-coumaric acid, diosmetin, eriodictyol, ethyl gallate, ferulic acid, flavanomarein, gallic acid (GA), gentisic acid, hyperoside, hesperidin, *p*-hydroxybenzoic acid, 2,4-dihydroxybenzoic acid, jasmonic acid, isorhamnetin, luteolin, naringin, protocatechuic acid, phloretin, pinoresinol, procyanidin B1, rutin, salicylic acid, sinapic acid, syringic acid, taxifolin, quercetin, quinic acid, vanillin, and vanillic acid.

### 2.2 Instruments

Gas chromatography–mass spectrometry (GC-MS) analyses were carried out using an Agilent 5975 GC-MSD system (Agilent Technologies, Santa Clara, CA). UHPLC-HRMS/MS analysis was performed using a Thermo Scientific Vanquish Flex UHPLC system paired with an Orbitrap Exploris 120 high-resolution mass spectrometer (Thermo Fisher Scientific, Bremen, Germany), equipped with a heated electrospray ionization (HESI) source. Absorbance values for spectrophotometric assays were measured with a microplate reader (PowerWave XS; BioTek Instruments, Winooski, VT). The spectrophotometer was operated according to the manufacturer’s guidelines, and absorbance was recorded at assay-specific wavelengths. Sample pipetting was performed using an Eppendorf Xplorer 12-channel electronic pipet (10–300 μL; Eppendorf, Hamburg, Germany). A 96-deep-well round-bottom polypropylene plate (2.2 mL) and a 96-well flat-bottom white polystyrene microplate (nonsterile; Greiner Bio-One, Kremsmünster, Austria) were used to handle samples and measure absorbance.

### 2.3 Plant Material

*M. aquifolium* samples, including the flowers, leaves, and fruits were collected from Günalan village, Ankara, Türkiye. The flowers and the leaves were harvested during the flowering period, while the fruits were collected at full ripeness during the fruiting stage. Immediately after collection, the fruits were frozen at −20 °C and subsequently lyophilized. Following lyophilization, the fruit pulp and seeds were manually separated. The seeds were thoroughly washed to remove any residual pulp, then air-dried in the shade at ambient temperature for 3 days and stored at +4 °C until further analysis. A voucher specimen (ESSE 16356) was deposited in the Herbarium of the Faculty of Pharmacy, Anadolu University, Eskisehir, Türkiye.

### 2.4 Extraction of Plant Material

Prior to extraction, the seeds, fruit pulp, flowers, and leaves of *M. aquifolium* were individually homogenized using a laboratory grinder. Each plant part was subjected to maceration with 70% aqueous ethanol at the room temperature under continuous shaking for 72 h. To minimize potential degradation of light-sensitive and thermolabile constituents, the maceration was performed in amber-colored Erlenmeyer flasks under light-protected conditions. In addition, the extraction solvent was refreshed after each extraction cycle to reduce prolonged exposure and minimize potential degradation during extended maceration. After decanting and filtration, the plant residues were re-extracted under the same conditions for an additional 72 h to ensure maximum yield. The combined supernatants were filtered through Whatman No. 1 filter paper. The ethanol phase was evaporated under reduced pressure using a rotary evaporator to yield crude dry extracts. Remaining aqueous fractions were lyophilized to complete solvent removal. All dried extracts were stored in amber glass bottles or sealed Eppendorf tubes at 4 °C until further analysis.

### 2.5 GC-FID/MS Analysis of Silylated Extracts

For the chemical composition analysis, the 70% (v/v) aqueous ethanol extracts of *M. aquifolium* were subjected to derivatization by silylation prior to GC-FID and GC-MS analysis. Precisely 2.0 mg of each dried extract was dissolved in 80 μL of pyridine and subsequently mixed with 80 μL of BSTFA containing 1% trimethylchlorosilane (TMCS). The reaction mixture was vortexed and heated at 100 °C for 1 h using a sand bath to ensure complete derivatization. After cooling to room temperature, 1.0 μL of the silylated solution was injected directly into the GC-FID and GC-MS instruments for qualitative and semiquantitative analysis.^21,22^

The silylated 70% (v/v) aqueous ethanol extracts of *M. aquifolium* were analyzed using an Agilent 7890A gas chromatograph coupled with a 5975C Inert Mass Selective Detector (MSD) featuring a Triple-Axis detector (Agilent Technologies, Santa Clara, CA). Chromatographic separation was achieved on an HP-5 FSC column (30 m × 0.25 mm i.d., 0.25 μm film thickness; Agilent Technologies, Wilmington, DE). Helium was used as the carrier gas at a constant flow rate of 0.8 mL/min. The oven temperature program was as follows: an initial temperature of 100 °C, ramped at 3 °C/min to 320 °C, followed by an isothermal hold at 320 °C for 16.67 min. Samples were injected in splitless mode with an injection port temperature of 250 °C. The FID temperature was maintained at 300 °C. The MS transfer line temperature was set at 280 °C. Mass spectra were acquired in electron impact (EI) mode at 70 eV over a scan range of 35–1050 *m*/*z*. The ion source temperature was maintained at 230 °C. Data were acquired at a scan rate of 2.5 scans/s. The broad mass range was intentionally selected to enable comprehensive detection of high-mass silylated polar metabolites formed after BSTFA derivatization. The applied scan rate represents a compromise between mass spectral coverage and chromatographic peak definition and was considered sufficient to provide an adequate number of data points per chromatographic peak under the selected GC conditions. Since the analytical focus of this study was on qualitative and semiquantitative profiling of derivatized polar constituents rather than high-frequency acquisition of narrow peaks, the selected acquisition parameters were deemed appropriate for the intended characterization. Compound identification was performed by comparing the obtained mass spectra and retention times with those of authentic standards and entries in the Wiley GC/MS Library (Wiley, New York, NY), and the in-house “Special Özek Silyl Derivative Library” developed specifically for silylated phytochemicals. Only matches with a similarity index above 90% and appropriate RRI from the literature published^21^ were accepted for library identification. Quantitative analysis was conducted by calculating the relative percentage of each peak based on FID chromatograms.^21,22^

### 2.6 UHPLC-HRMS/MS Orbitrap Phenolic Compounds Analysis

The evaluation of *M. aquifolium* extracts, which consist of 70% (v/v) aqueous ethanol, was conducted using UHPLC-HRMS/MS to analyze phenolic compounds and other secondery metabolites. Analyses were conducted using a Thermo Scientific Dionex Ultimate 3000 RS UHPLC system and a Q Exactive quadrupole-Orbitrap hybrid mass spectrometer (Thermo Fisher Scientific) with an HESI source. At 40 °C, chromatographic separation was obtained using an Acquity BEH C18 column (100 × 2.1 mm, 1.7 μm particle size; Waters) maintained at 40 °C in a thermostated column oven. In the mobile phase, solvent A (water with 0.1% formic acid) and solvent B (methanol with 0.1% formic acid) were delivered a flow rate at 0.5 mL/min. The gradient program consisted of the following intervals: 0–1 min at 5% B, 1–10 min with a linear increase to 100% B, 10–12 min held at 100% B, and 12–15 min for re-equilibration back to 5% B. The injection volume was 5 μL. Data-dependent acquisition (Top5) was used to run the mass spectrometer in negative ionization mode. Negative mode was selected because phenolic compounds ionize more efficiently under these conditions. Full MS scans were acquired at a resolution of 70.000 (FWHM at m/z 200), and MS/MS scans at 17.500. HESI parameters: spray voltage −3.0 kV, capillary 320 °C, probe heater 400 °C, sheath gas 60, auxiliary gas 25 (arbitrary units), and S-lens RF level 50. Data was processed using TraceFinder 5.1 using the standrad HRAM. Compound identification relied on retention duration, precise mass (within 5 ppm), MS/MS fragmentation patterns, and isotopic distribution. Compound confirmation was done using an in-house database of 87 authentic phenolic standards. Authentic standards were injected under identical chromatographic conditions to verify retention time and fragmentation consistency. Only hits with an isotopic pattern match score above 80% were valid.^23^

Silylation-based GC-FID/MS facilitates the identification of semipolar, thermally stable metabolites, such as organic acids, sterols, phenolics and sugars. UHPLC-HRMS/MS provides high-resolution identification of phenolic chemicals and polar secondary metabolites. These two complementary platforms offer orthogonal coverage and mitigate metabolite loss resulting from ionization or volatility constraints.

### 2.7 Determination of Total Phenolic and Flavonoid Contents

Total phenolic content (TPC) was determined by the Folin–Ciocalteu method using gallic acid as the standard.^24^ Absorbance was measured at 765 nm, and results were expressed as mg gallic acid equivalents per gram of extract (mg GAE/g_extract_). The quantification was based on a gallic acid calibration curve (*y* = 0.8421*x* + 0.077, *R* ^2^ = 0.9993).

Total flavonoid content (TFC) was assessed by the aluminum chloride colorimetric method with rutin as the standard.^25,26^ Absorbance was measured at 415 nm, and values were expressed as mg rutin equivalents per gram of extract (mg RE/g_extract_). A rutin calibration curve (*y* = 0.1753x, *r* ^2^ = 0.9939) was used for quantification.

### 2.8 Free Radical Scavenging Assay (DPPH Test)

The free radical scavenging capacity of the extracts was evaluated using the DPPH assay, based on a modified version of the method developed by Brand-Williams et al.^27^ Samples for extraction and reference were dissolved in methanol with 10% DMSO. The samples were combined with DPPH solution in a 96-well microplate and incubated in the absence of light for 30 min. Absorbance was recorded at 517 nm, utilizing gallic acid as the reference antioxidant. Experiments were conducted in triplicate. The percentages of radical scavenging and IC_50_ values were obtained from the concentration–response curves. *SigmaPlot* (Version 15.0) was employed for data analysis.

### 2.9 Lipid Peroxidation Inhibition Assay (β-Carotene Bleaching Test)

The ability of the extracts to inhibit lipid peroxidation was evaluated using a modified β-carotene-linoleic acid bleaching assay.^28^ The emulsion was produced with β-carotene, linoleic acid, and Tween-20 after the chloroform was evaporated under decreased pressure. Oxygenated water was then added to create a stable emulsion, which was made fresh before each examination. Butylated hydroxyanisole (BHA) served as the reference antioxidant. In a 96-well plate, the sample or reference solutions were combined with the emulsion, and absorbance was quantified at 492 nm for 105 min at 50 °C. The antioxidant activity was measured as the percentage of suppressed β-carotene discoloration relative to the control.

### 2.10 Oxygen Radical Absorbance Capacity (ORAC) Assay

The ORAC assay was performed with slight modifications based on previously published protocols.^28^ For each reaction, 50 μL of sample solution or Trolox standard, 100 μL of fluorescein solution (45 nM), and 50 μL of 2,2′-azobis­(2-methylpropionamidine) dihydrochloride (AAPH, 15 mM) were pipetted into the wells of a 96-well microplate. The fluorescence was measured kinetically every minute for 60 min at 37 °C using a multimode microplate reader (Synergy HT, BioTek Instruments), with an excitation wavelength of 485 nm and an emission wavelength of 528 nm. Trolox was used as the standard antioxidant, and calibration curves were constructed using a series of concentrations ranging from 0.5 to 9.5 μM. All measurements were carried out in quadruplicate. ORAC values were calculated by comparing the area under the fluorescence decay curve (AUC) of the samples against that of the blank. Results were expressed as millimoles of Trolox equivalents per gram of extract (μmol TE/g_extract_).

### 2.11 Trolox Equivalent Antioxidant Capacity (TEAC) Assay

The antioxidant capacity of the extracts was evaluated using the ABTS^+•^ radical scavenging assay, with results expressed as Trolox equivalents, as previously described.^28^ A stock solution of 7 mM ABTS was prepared by dissolving 6.6 mg of potassium persulfate in 10 mL of distilled water and allowing the mixture to incubate in the dark for 16 h to generate ABTS^+•^ radicals. Prior to use, the resulting solution was diluted with absolute ethanol to achieve an absorbance of 0.700–0.800 at 734 nm. Extract and Trolox stock solutions were prepared in methanol containing 10% DMSO at a concentration of 2 mg/mL for the extracts, and between 0.125 and 3.0 mM for Trolox. For the assay, 10 μL of each sample or standard was added to 990 μL of the ABTS^+•^ working solution in a 96-deep-well plate. The mixture was incubated in the dark at room temperature for 30 min, after which the absorbance was measured at 734 nm using a microplate reader. Antioxidant activity was expressed as Trolox equivalent antioxidant capacity (TEAC), calculated from a standard calibration curve for Trolox (*y* = 24.976*x* + 2.6357; *r* ^2^ = 0.9993). All measurements were performed in quadruplicate, and results were reported as μmol TE/g_extract_, along with their corresponding standard errors.

### 2.12 Cupric Ion Reducing Antioxidant Capacity (CUPRAC) Assay

The cupric ion-reducing capacity of the extracts was determined using the CUPRAC method originally described by Apak et al.,^29^ with slight modifications. Extracts were dissolved in a 10% DMSO–methanol mixture. CuCl_2_ solution (1.0 × 10^–2^ M) and ammonium acetate buffer (1.0 M, pH 7.0) were prepared in ultrapure water, while the neocuproine (Nc) solution (7.5 × 10^–3^ M) was prepared in absolute ethanol. For each reaction, 55 μL of the extract or standard solution, 50 μL of CuCl_2_ solution, 50 μL of neocuproine solution, and 50 μL of ammonium acetate buffer were pipetted into wells of a 96-well microplate. The control wells contained 10% DMSO–methanol instead of extract solution. The mixtures were incubated at 25 °C for 30 min. Absorbance was then measured at 450 nm using a Biotek Powerwave XS microplate reader (BioTek Instruments). Trolox was used as the reference antioxidant, and a calibration curve was constructed using the following linear equation: *y* = 1.069*x* + 0.1341 (*r* ^2^ = 0.9986), where y is the absorbance and *x* is the concentration of Trolox equivalents. Results were expressed as millimoles of Trolox equivalents per gram of extract (mmol TE/g_extract_) and reported as mean ± standard deviation (SD).

### 2.13 α-Amylase Inhibitory Activity Assay

The inhibitory effect of the extracts on α-amylase activity was evaluated using the I_2_/KI colorimetric method, with slight modifications based on a previously described protocol.^30^ Acarbose was utilized as the standard inhibitor. Extracts were produced in methanol with 10% DMSO. Equal volumes of the sample and enzyme solutions were incubated at 37 °C in a 96-well plate, followed by the addition of the starch substrate. After a subsequent incubation, the reaction was terminated with HCl and processed using I_2_/KI reagent. Absorbance was assessed at 630 nm, and inhibition was evaluated in comparison to the control wells.

### 2.14 Cell Culture and Treatments

NIH/3T3 mouse fibroblast cells (ATCC, CRL-1658) were cultured in Dulbecco’s Modified Eagle’s Medium (DMEM) supplemented with 10% fetal bovine serum (FBS) and 1% penicillin–streptomycin until they reached 70–80% confluence. Similarly, human monocyte THP-1 cells (ATCC, TIB-202) were maintained in Roswell Park Memorial Institute (RPMI) medium supplemented with 10% FBS, 1% penicillin–streptomycin, and 0.05 mM 2-mercaptoethanol (Gibco, 2198–023) under 5% CO_2_ at 37 °C until 70–80% confluence was achieved. THP-1 monocytes were differentiated into M0 macrophages by incubation with 100 ng/mL phorbol 12-myristate 13-acetate (PMA) in RPMI-1640 medium supplemented with 1% FBS for 24 h, followed by a 48 h resting period in PMA-free RPMI medium containing 10% FBS to allow macrophage maturation.^31,32^

Prior to the experimental procedures, cells were stained with Trypan Blue, counted using a cell counter (Cedex, Roche), and experiments were performed using appropriate viable cell densities.

### 2.15 Cytotoxic Effects of Extracts on THP-1 (M0) and NIH/3T3 Cells

The cytotoxic potential of *M. aquifolium* was evaluated using the MTT (3-(4,5-Dimethylthiazol-2-yl)-2,5-diphenyltetrazolium bromide) colorimetric assay in 96-well plates on THP-1 (M0) and NIH/3T3 cell lines. This assay determines cell viability based on the conversion of the yellow tetrazolium salt (MTT) into purple formazan crystals by metabolically active cells. The resulting color intensity, measured spectrophotometrically, reflects cellular metabolic activity and proliferation, with higher absorbance values indicating greater viability (Haffani et al.). For the assay, cells were seeded at a density of 1 × 10^4^ cells per well and allowed to adhere for 24 h. They were then exposed to different concentrations of the test samples (1, 10, 100, and 400 μg·mL^–1^) for 24 and 48 h. Following treatment, 10 μL of MTT reagent was added to each well and incubated for 3 h. Absorbance was recorded at 540 nm using a Cytation 3 Cell Imaging Multi-Mode Reader (BioTek). Cell viability was calculated as a percentage relative to untreated control cells.^33–35^

## 3 Results and Discussion

### 3.1 Extraction Yields

The present study offers a detailed phytochemical and biological investigation of *M. aquifolium*, addressing existing gaps in the literature through comparative analysis of various plant parts. Separate extractions were performed on the flowers, leaves, fruit pulp, and fruit seeds using 70% (v/v) aqueous ethanol, and the corresponding extract yields and extract amounts are summarized in Table 1.

**Table 1:** Extraction Yields of *M. aquifolium* Extracts Prepared with 70% (v/v) Aqueous Ethanol

extracts	amounts of extract, g	yield^a^, %
Flower extract (FE)	4.51	22.50
Leaf extract (LE)	22.12	13.83
Fruit pulp extract (FPE)	27.32	19.80
Fruit seeds extract (FSE)	13.5	10.14

a Yield was calculated based on the air-dried plant weight of the plant material.

The extraction methodology utilized in this investigation was carefully planned to ensure comprehensive recovery of polar and color-associated metabolites, which was crucial for comparative, organ-specific profiling. Consequently, maceration persisted until a noticeable depletion of color intensity in the extraction fluid was observed. Since all plant organs were handled under uniform, light-protected conditions, the resultant metabolite profiles indicate inherent chemical disparities across the organs rather than technical inconsistencies.

### 3.2 GC-FID/MS Analysis of Silylated Extracts

The polar constituents of *M. aquifolium*, extracted using 70% (v/v) aqueous ethanol, were derivatized with BSTFA containing 1% TMCS to enhance volatility and thermal stability prior to GC-FID/MS analysis. The resulting silyl derivatives were analyzed on an apolar HP-5 capillary column using an optimized temperature program to ensure effective chromatographic separation. Retention characteristics were established using a homologous series of *n*-alkanes (C_8_–C_40_) as external standards for the calculation of relative retention indices (RRIs).

Qualitative assessment was based on a combined evaluation of mass spectral fragmentation patterns, retention behavior (RRIs), and comparison with reference data from commercial libraries (NIST, Wiley) and published studies.^21^ Compound identification and characterization were carried out using complementary GC-FID/MS data, including mass spectral fragmentation patterns and relative retention indices. Quantitative assessment was based on FID peak areas without applying normalization procedures. All the identified components are presented in Table 2.

**Table 2:** GC-FID/MS Analysis Results of Silylated 70% (v/v) Aqueous Ethanol Extracts of *M. aquifolium* Leaves, Flowers, Fruit Pulps and Seeds^a^,^b^

			70% (v/v) aqueous ethanol extracts			
			leaf	flower	fruit Pulp	seeds
RRI^c^	RRI^d^	compound	%	%	%	%
1180	1174	l-Proline-TMS		0.10 ± 0.00		
1234	1239	Ethyl phosphoric acid-2 TMS		0.09 ± 0.00		
1281	1293	Glycerol-3 TMS				0.56 ± 0.03
1284	1289	Phosphoric acid-3 TMS	0.11 ± 0.00	0.33 ± 0.02		
1387		Erythronic acid γ-lactone −2 TMS^e^		0.14 ± 0.01		
1506	1512	Malic acid-3 TMS	1.67 ± 0.10	1.06 ± 0.01	17.50 ± 0.01	4.67 ± 0.32
1585		l-Threonic acid-4 TMS (isomer-2)^e^		0.11 ± 0.00		
1738	1740	D-Xylopyranose-4 TMS		0.09 ± 0.00		
1797		Tagatofuranose-5 TMS (isomer-2)^e^	0.24 ± 0.01	0.09 ± 0.00		
1809	1817	α-d-Methylfuranoside-4 TMS				0.69 ± 0.00
1840	1843	Shikimic acid-4 TMS	0.08 ± 0.00			
1844	1843	α-Fructofuranose-5 TMS	0.17 ± 0.00	1.32 ± 0.04	4.57 ± 0.12	6.11 ± 0.33
1852	1854	β-Fructofuranose-5 TMS	0.23 ± 0.01	1.83 ± 0.07	8.52 ± 0.11	6.90 ± 0.21
1856		Ethyl 2.3.4.6-tetrakis-O-(trimethylsilyl)-d-glucopyranoside^e^				1.38 ± 0.07
1858	1887	Fructopyranose-5 TMS	0.08 ± 0.00	1.09 ± 0.11		
1867		Xylose derivative^e^				1.01 ± 0.01
1873		Furanose derivative^e^				2.43 ± 0.13
1885	1887	β-Glucofuranose-5 TMS		0.22 ± 0.00		1.00 ± 0.05
1898	1900	Quinic acid-5 TMS	4.77 ± 0.03	5.45 ± 0.19	7.44 ± 0.17	2.51 ± 0.17
1907	1924	Lidocaine		0.21 ± 0.00		
1914	1915	Syringic acid-2 TMS	0.92 ± 0.06	0.21 ± 0.01		
1929	1930	α-Glucopyranose-5 TMS	1.83 ± 0.11	5.37 ± 0.20	13.17 ± 0.21	4.59 ± 0.28
1939	1945	β-Galactopyranose-5 TMS				1.35 ± 0.11
1942	1943	β-Mannopyranose-5 TMS				0.72 ± 0.03
1984	1979	Ascorbic acid-4 TMS	0.24 ± 0.01			
1986		γ-Lactone saccharic acid-4 TMS^e^			1.71 ± 0.02	
1987		Glucaric acid γ-lactone-4 TM^e^	1.17 ± 0.07	1.44 ± 0.13		
2009		Glucaric acid γ-lactone-6 TMS^e^	0.78 ± 0.00	0.50 ± 0.00		
2023	2032	β-Glucopyranose-5 TMS	1.19 ± 0.02	5.32 ± 0.24	13.42 ± 0.14	5.69 ± 0.03
2046	2046	Gluconic acid-6 TMS			0.87 ± 0.02	
2049	2052	Hexadecanoic acid (=Palmitic acid)-TMS	0.12 ± 0.00	0.10 ± 0.00		
2113	2113	Methoxycyclohexanepentol-5 TMS (isomer-3)	0.07 ± 0.00			
2127	2129	myo-Inositol-6 TMS	0.93 ± 0.04	3.11 ± 0.13		
2151	2154	(*E*)-Caffeic acid-3 TMS	4.21 ± 0.17	0.08 ± 0.00		
2221	2222	(*Z*)-9-Octadecenoic acid (=Oleic acid)-TMS		0.10 ± 0.00		
2250	2250	Octadecanoic acid (=Stearic acid)-TMS		0.07 ± 0.00		
2448	2448	Eicosanoic acid (=Arachidic acid)-TMS		0.13 ± 0.00		
2591		Sucrose-7 TMS derivative^e^	0.20 ± 0.00	0.14 ± 0.00		1.84 ± 0.11
2650		Sucrose derivative^e^				3.03 ± 0.14
2696		Sucrose derivative^e^				1.34 ± 0.05
2700		Sucrose derivative^e^				2.97 ± 0.01
2706		Sucrose derivative^e^				1.53 ± 0.09
2708		Cyclohexanedicarboxylic acid -TMS derivative^e^	0.78 ± 0.03	0.76 ± 0.00		
2713	2714	Sucrose-8 TMS	34.18 ± 0.26	11.55 ± 0.36		
2740		Cyclohexanedicarboxylic acid -TMS derivative^e^	0.19 ± 0.00			
2743	2744	Maltose-8 TMS (isomer-1)				0.52 ± 0.02
2757		Cyclohexanedicarboxylic acid -TMS derivative^e^		0.10 ± 0.00		
2764		Cyclohexanedicarboxylic acid -TMS derivative^e^		0.09 ± 0.00		
2782		Maltose derivative^e^				7.03 ± 0.02
2804	2808	1-Monostearin-2 TMS		0.11 ± 0.00		
2826		Solidroside-5 TMS^e^	0.52 ± 0.00	0.24 ± 0.00		
2833		Cyclohexanedicarboxylic acid -TMS derivative^e^	0.19 ± 0.01			
2844	2849	neo-Trehalose-8 TMS		0.11 ± 0.00	0.62 ± 0.01	
2935	2936	Catechin-5 TMS				0.69 ± 0.04
3004	3005	β-Isomaltose-8 TMS	0.23 ± 0.00			
3042	3043	Hexacosanoic acid-TMS			1.89 ± 0.08	
3060	3058	Chlorogenic acid-6 TMS		0.28 ± 0.01		
3076	3077	Nonacosan-10-ol-TMS	0.56 ± 0.00			
3244	3248	crypto-Chlorogenic acid-6 TMS	27.94 ± 0.15	23.52 ± 0.32	2.27 ± 0.08	
3264	3269	5-Caffeoylquinic acid-6 TMS	0.17 ± 0.01	0.21 ± 0.00		
3275	3253	Campesterol-TMS		1.46 ± 0.02		
3368	3345	β-Sitosterol-TMS	0.40 ± 0.00	1.05 ± 0.09	0.52 ± 0.00	
3386		Raffinose derivative^e^		11.04 ± 0.18		2.59 ± 0.20
3507	3505	Raffinose-11 TMS	0.20 ± 0.01	0.22 ± 0.00		

a ≥0.5%

b %: Calculated from the FID chromatogram

c RRI: Relative retention indices calculated against *n*-alkanes for apolar column

d RRI: Relative retention indices from literature^21^

e Tentative identification from Wiley-NIST Library

Silylation is a commonly employed derivatization strategy in GC-MS applications, particularly useful for analyzing compounds with polar functional groups.^36^ This method modifies low-volatility moieties such as hydroxyl (−OH), carboxyl (−COOH), and amino (−NH_2_) groups by converting them into thermally stable, more volatile silyl derivatives. Such transformation enhances peak sharpness, improves chromatographic resolution, and allows for more accurate quantification, thereby enabling efficient analysis of polar compounds in complex matrices.^37^ Silylation enhances the volatility, thermal stability, and chromatographic behavior of polar compounds, improving sensitivity and enabling the detection of metabolites.^38–42^ The GC-FID/MS analysis of silylated polar extracts demonstrated distinct chemical profiles of the different plant parts. In the leaf extract, sucrose (34.18 ± 0.26%) and *crypto*-chlorogenic acid (27.94 ± 0.15%) were identified as the principal constituents. Similarly, the flower extract exhibited *crypto*-chlorogenic acid as the dominant compound (23.52 ± 0.32%), followed by sucrose (11.55 ± 0.36%). In the fruit pulp extract, malic acid (17.50 ± 0.01%) and β-d-glucopyranose (13.42 ± 0.14%) emerged as the most abundant components. As for the seed extract, a maltose-derived silyl compound (7.03 ± 0.02%) was found to be the predominant constituent. These findings indicate considerable variation in polar metabolite distribution among different plant parts of *M. aquifolium*, emphasizing the importance of organ-specific phytochemical profiling.

To the best of our knowledge, this study represents the first silylation-based GC-FID/MS investigation of the polar extracts from various organs of *Mahonia* species. Among the identified constituents, sucrose and maltose—key plant sugars involved in energy storage, transport, and starch metabolism—were abundant, particularly in the leaf and seed extracts.^43–45^ Malic acid, a primary organic acid contributing to taste, cellular respiration, and stress signaling, was dominant in the fruit pulp extract.^46–48^ *Crypto-*chlorogenic acid, a structural isomer of chlorogenic acid, was the most abundant phenolic compound detected in both leaf and flower extracts. This compound is well-documented for its antioxidant and protective effects against oxidative stress.^49–51^ These findings highlight the biochemical richness of *M. aquifolium* and offer novel insight into its polar phytochemical composition.

### 3.3 UHPLC-HRMS/MS Orbitrap Phenolic Compounds Analysis

In this study, both qualitative and quantitative analyses of the phenolic compounds were conducted on the 70% (v/v) aqueous ethanol extracts of *M. aquifolium* leaves, flowers, fruit pulp, and seeds. A wide range of the phenolic constituents were identified and quantified using UHPLC-HRMS/MS, including 3-hydroxytyrosol, 4-hydroxybenzoic acid, 4-*O*-caffeoylquinic acid, apigenin-7-*O*-glucoside, caffeic acid, catechin, chlorogenic acid, *p*-coumaric acid, diosmetin, flavanomarein, gentisic acid, isorhamnetin, luteolin, naringin, and vanillin. Qualitative identification of the compounds was performed based on retention time, exact mass, and MS/MS fragmentation patterns. Quantitative analysis was carried out using external calibration, with calibration curves generated for each analyte using a 12-point serial dilution of a standard mixture composed of commercially available reference compounds. The concentrations of the identified phenolic compounds were calculated accordingly. Detailed identification and quantification results are provided as tabulated data in the Supporting Information (Table S1–S4). A concise summary of the quantified phenolic compounds is provided in Table 3 to facilitate comparison among different plant organs.

**Table 3:** Composition of the Phenolic Compounds in *M. aquifolium* Polar Extracts (mg/g_dry extract_)­^a^

	70% (v/v) aqueous ethanol extracts			
compounds	leaf	flowers	fruit pulp	seeds
3-Hydroxytyrosol	1.20 ± 0.11	19.15 ± 0.53		4.64 ± 0.01
4-Hydroxybenzoic acid	493.1 ± 0.00	1.88 ± 0.01		0.45 ± 0.01
4-*O*-Caffeoylquinic acid	27.67 ± 0.12	92.28 ± 2.06		
Caffeic acid	20.65 ± 0.25	12.35 ± 0.27	0.18 ± 0.01	3.73 ± 0.03
Chlorogenic acid	527.74 ± 8.22	786.06 ± 5.05	13.32 ± 0.01	121.17 ± 3.09
Gentisic acid				
*p*-Coumaric acid	0.12 ± 0.00	1.22 ± 0.05		0.17 ± 0.01
Protocatechuic acid	8.95 ± 0.10	18.81 ± 0.12	0.27 ± 0.00	7.65 ± 0.18
Apigenin-7-*O*-glucoside	11.00 ± 0.80			1.38 ± 0.02
Catechin			1.11 ± 0.06	47.80 ± 0.23
Diosmetin	3.13 ± 0.19	0.58 ± 0.01		0.89 ± 0.01
Flavanomarein				46.18 ± 0.57
Isorhamnetin	0.10 ± 0.00	3.63 ± 0.04		0.12 ± 0.01
Luteolin	38.78 ± 0.74	3.99 ± 0.07	0.76 ± 0.04	12.05 ± 0.14
Naringin	0.19 ± 0.00			
Vanillin		3.09 ± 0.00		

a dw: dry weight.

Chlorogenic acid was the most abundant phenolic compound identified in all 70% (v/v) aqueous ethanol extracts of *M. aquifolium*, with concentrations ranging from 13.32–786.06 mg/g_dry extract_. The highest level was observed in the flower extract (786.06 ± 5.05 mg/g_dry extract_), while the lowest was recorded in the fruit pulp extract (13.32 ± 0.01 mg/g_dry extract_). In the flowers extract, 4-O-caffeoylquinic acid (92.28 ± 2.06 mg/g_dry extract_) was the second most abundant compound. In the leaf extract, 4-hydroxybenzoic acid (493.10 ± 0.00 mg/g_dry extract_) followed chlorogenic acid in concentration. In the fruit pulp 70% ethanol extract, catechin (1.11 ± 0.06 mg/g_dry extract_) was the second most abundant phenolic compound. In the fruit seed extract, catechin (47.80 ± 0.23 mg/g_dry extract_) and flavanomarein (46.18 ± 0.57 mg/g_dry extract_) were also detected at significant levels. Additionally, 3-hydroxytyrosol was found in the flowers (19.15 ± 0.53 mg/g_dry extract_), leaf (1.20 ± 0.11 mg/g_dry extract_), and fruit seed extracts (4.64 ± 0.01 mg/g_dry extract_). Among the identified phenolic compounds, several flavonoids—including apigenin-7-*O*-glucoside, diosmetin, isorhamnetin, luteolin, and naringin—were consistently identified in 70% (v/v) aqueous ethanol extracts. The findings underscore the existence of structurally varied flavonoids in the polar fractions and illustrate that UHPLC-HRMS/MS facilitates thorough detection of thermolabile and less volatile components, which may be undervalued by GC-MS-based silylation methods.^52,53^

Importantly, the pronounced enrichment of 3-hydroxytyrosol in flower extracts, reaching levels approximately 4-fold higher than in other plant organs, highlights a clear organ-specific accumulation pattern.^54,55^ Such enrichment may be associated with the protective role of phenolic alcohols in floral tissues, which are exposed to increased oxidative and environmental stress during reproductive development.^56,57^ Similarly, the exclusive detection of vanillin in flowers, naringin in leaves, and flavanomarein in seeds suggests highly regulated, organ-dependent phenolic biosynthesis.^58–60^ Vanillin may be linked to aromatic and signaling functions in reproductive organs, whereas naringin accumulation in leaves is consistent with its reported roles in UV protection^61^ and herbivore defense.^62,63^ The presence of flavanomarein exclusively in seeds may reflect its function in phenolic storage or protection of embryonic tissues.^64–66^

These organ-specific distribution patterns were consistently supported by UHPLC-HRMS/MS data and corroborated by complementary GC-FID/MS analyses, reinforcing that the observed chemical differences reflect inherent biological specialization rather than analytical variability.

According to literature, the ethanolic leaf extract of *M. aquifolium* contained high levels of phenolic compounds. Among the hydroxycinnamic acid derivatives, chlorogenic acid was found in the highest concentration (5049 ± 25 μg/mL), followed by ferulic acid (11.8 ± 1.9 μg/mL) and *p-*coumaric acid (4.3 ± 1.0 μg/mL). Additionally, two flavonoid glycosides—rutin and isoquercitrin—were detected at significant concentrations in the leaf extract (371 ± 18 μg/mL and 217 ± 7.6 μg/mL, respectively). In contrast, quercetin levels were below the detection limit in all samples.^19^ In an earlier study by Andreicuţ et al., the highest concentration of chlorogenic acid was found in the flower ethanol extract (2013 ± 2 μg/mL). Additionally, *p*-coumaric acid was reported in the fruit ethanol extract (10.0 ± 0.3 μg/mL). The same study also confirmed the presence of rutin (73 ± 1.6 μg/mL) and isoquercitrin (29.7 ± 0.7 μg/mL) in the methanolic fruit extract.^67^ Other studies have also demonstrated that chlorogenic acid is the most abundant phenolic acid in *Mahonia* berries and leaves, with concentrations reaching up to 373 mg/100 g_fresh weight_ in *M. aquifolium* and ranging from 9.93 to 13.77 mg/g in *M. jaunsarensis* Ahrendt. Other phenolic acids identified in *Mahonia* species include caffeic acid, syringic acid, ferulic acid, *p*-coumaric acid, and gentisic acid, with their concentrations reported to vary during fruit ripening.^3,10^ Flavonoids such as quercetin-3-O-glucoside and isorhamnetin-3-O-glucoside are found in *M. bealei* (Fortune) Carr. leaves.^68^ In previous studies on *M. aquifolium*, the fruits have generally been examined as a whole, and the fruit pulp and seeds have not been analyzed separately. To date, no study has focused on the phenolic composition of polar extracts derived individually from the pulp and seed parts. Our study fills this gap by being the first to conduct both qualitative and quantitative analyses of polar extracts obtained separately from the fruit pulp and seeds. Furthermore, several compounds—flavanomarein, 3-hydroxytyrosol, and apigenin-7-*O*-glucoside—which had not been previously reported in the polar extracts of *M. aquifolium*, were identified and quantified for the first time in this work. These findings emphasize the distinct chemical profiles of different plant organs and support the relevance of organ-specific extraction approaches.

Consistent with previous findings, chlorogenic acid was identified as the predominant phenolic compound across all 70% (v/v) aqueous ethanol extracts, reinforcing its recognized role in the phytochemical profile and bioactivity of *M. aquifolium*. This outcome is particularly significant given the established cardiometabolic benefits of chlorogenic acid, including its capacity to reduce circulating levels of triglycerides.^69^ Notably, in contrast to earlier studies that reported ferulic acid, our analysis did not reveal its presence—neither qualitatively nor quantitatively—in any of the polar extracts examined. This absence may be attributed to differences in extraction techniques, plant organ specificity, or variations in environmental and geographical factors that influence phytochemical accumulation.^70,71^

Our study found 3-hydroxytyrosol in a 70% (v/v) aqueous ethanol extracts of the seeds, leaves, and flowers of the *M. aquifolium*, which is different from what earlier research reported. Found in olive oil and wine, 3-hydroxytyrosol is a naturally occurring antioxidant metabolite of dopamine.^72^ Additionally, our investigation found flavanomarein only in the fruit seed extract, in contrast to previous studies on *Mahonia* extracts. This molecule is well-known for its antioxidant, anti-inflammatory, neuroprotective, antidiabetic, and cardiometabolic activities, primarily due to its ability to control oxidative stress and inflammation-related signaling pathways. Finding 3-hydroxytyrosol and flavanomarein in the fruit seed polar extract shows a new discovery about plant chemicals and points to potential future research in medicine.^73,74^ Beyond individual compound identification, the pronounced organ-specific phenolic differentiation observed in *M. aquifolium* carries important implications for its practical utilization. The distinct enrichment patterns of bioactive compounds across flowers, leaves, fruit pulp, and seeds suggest that nonselective, whole-plant exploitation may not fully capture the functional potential of the species. Instead, the data support an organ-targeted utilization strategy, in which specific plant parts are selected based on their dominant phytochemical profiles and intended applications. From a food science perspective, the high chlorogenic acid and phenolic alcohol content of flowers may favor their use as antioxidant-rich functional ingredients, whereas the seed-specific accumulation of flavanomarein highlights the potential of seeds as a concentrated source of cardiometabolic and neuroprotective compounds. In pharmaceutical and nutraceutical contexts, such chemical specialization enables more rational extraction, formulation, and standardization strategies, reducing matrix complexity and improving reproducibility. Overall, these findings emphasize that organ-resolved phytochemical profiling is essential for optimizing processing strategies and maximizing the biological and functional value of *M. aquifolium*.

### 3.4 Total Phenolic, Flavonoid Contents and Biological Activity Results

To evaluate the antioxidant capacity attributed to phenolic constituents in natural products, the total phenolic content of the extracts was assessed using the Folin–Ciocalteu colorimetric method. The results were expressed in terms of gallic acid equivalents (GAE). Additionally, the total flavonoid content was measured via spectrophotometric analysis using the aluminum chloride (AlCl_3_) method, and the outcomes were expressed as rutin equivalents (RE). The results are shown in Figure 1.

**Figure 1:**
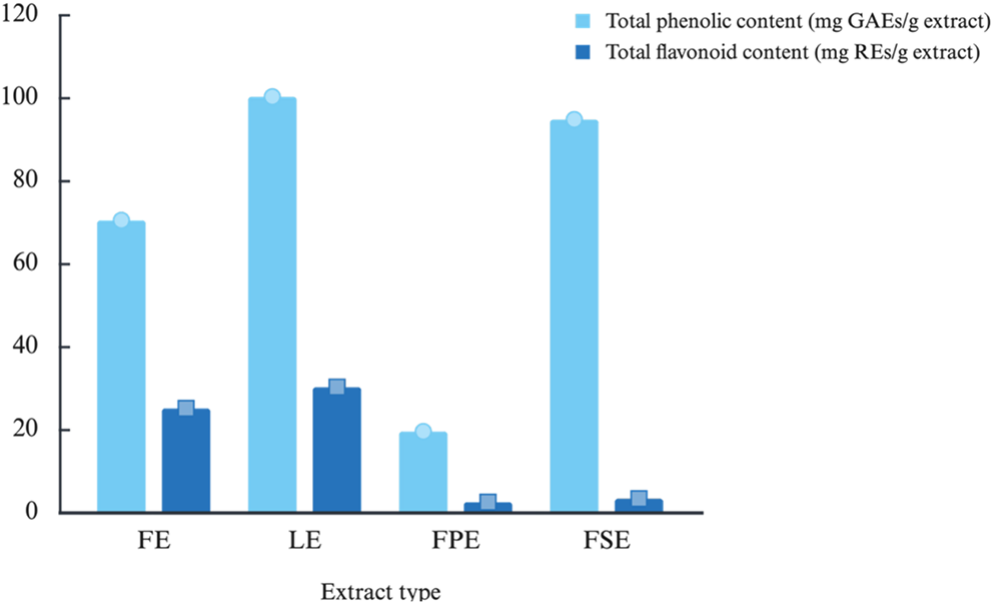
Total phenolic (mg GAE/g extract) and total flavonoid (mg RE/g extract) contents of 70% (v/v) aqueous ethanol extracts from *M. aquifolium* flowers (FE), leaves (LE), fruit pulp (FPE), and fruit seeds (FSE).

Our investigation showed that leaf 70% (v/v) aqueous ethanol extract has the highest phenolic (100.16 ± 1.90 mg GAEs/g_extract_) and flavonoid content (30.14 ± 0.02 mg REs/g_extract_). This finding aligns with the UHPLC-HRMS/MS study of phenolic chemicals reported in Table 3.

Flavonoids are known to exist in plant matrices as either free aglycones or glycosylated derivatives, prompting the intentional use of various reference standards in total flavonoid assays to represent this structural diversity.^75^ Rutin is frequently utilized as a reference compound for glycosylated flavonoids, while aglycone standards like quercetin or catechin are employed to assess free flavonoid forms.^76–78^ The total flavonoid content assay in this work was employed to offer a comparative evaluation of flavonoid prevalence across various plant organs, rather than an absolute measurement of specific flavonoid compounds.^79^

A previous study reported the total phenolic and flavonoid contents in a study using 80% methanol extract of *M. aquifolium* fruits were recorded as 806.19 ± 6.44 mg GAE/100 g_fresh mass_ and 78.17 ± 1.81 mg RE/100g_fresh mass_ respectively.^80^ Coklar et al. reported about the total phenolic content of 1014.60 ± 4.32 mg GAE/100 g_fresh weight_ in ethanol extracts prepared from whole *M. aquifolium* berries.^3^ Bisht et al. (2023) reported that aqueous ethanol extracts of *M. jaunsarensis* collected from different geographical locations exhibited total phenolic contents ranging from 1.56 ± 0.01 to 1.80 ± 0.01 mg GAE/g, and total flavonoid contents between 1.49 ± 0.01 and 1.73 ± 0.02 mg RE/g.^10^ In contrast, the present study focused on 70% ethanol extracts from individually separated plant parts, with total phenolic content ranging from 19.43 ± 1.32 to 100.16 ± 1.90 mg GAE/g_extract_. Additionally, the 70% aqueous ethanol extracts in the present study demonstrated significantly higher total phenolic contents, with values reaching up to 100.16 ± 1.90 mg GAE/g_extract_, particularly in the leaf and fruit seed extracts. While the flavonoid content remained low or nondetectable in most samples, the fruit seed extract yielded a measurable amount (1.22 ± 0.01 mg RE/g_extract_), which is within the same range as those reported for *M. jaunsarensis*. These findings suggest that *M. aquifolium* may offer a more phenolic-rich profile under similar extraction conditions, especially when organ-specific analysis is considered.^10^ These results show that several organs, especially leaf and fruit seed, also show significant phenolic content even if the values are not exactly equivalent due of variances in sample preparation and expression units. This enhances the possibilities of focused extraction methods in optimizing phytochemical output from plant sections.^81^

Using several *in vitro* assays, including the DPPH radical scavenging assay, oxygen radical absorbance capacity (ORAC), β-carotene bleaching assay, and cupric ion reducing antioxidant capacity (CUPRAC), the antioxidant potential of 70% (v/v) aqueous ethanol extracts obtained from the flowers, leaves, fruit pulp, and fruit seeds of *M. aquifolium* was evaluated. These assays were employed as complementary tools to provide a relative comparison of antioxidant responses among different plant organs rather than as absolute measures of antioxidant efficacy.^82–84^ In addition, the α-amylase inhibitory activity was assessed as a preliminary indicator of potential enzyme interaction. The results of the evaluated antioxidant, enzyme inhibitory, and cytotoxic activities are summarized in Table 4.

**Table 4:** Biological Activity of *M. aquifolium* 70% (v/v) Aqueous Ethanol Extracts^f^

extract type	DPPH^a^ (IC_50_, μg/mL)	TEAC (TE, mM)	CUPRAC (TE, mM)	ORAC^b^ (μmol TE/g_extract_)	β-carotene bleaching test, (IC_50_, μg/mL)	α-amylase inhibition, (IC_50_, μg/mL)	cytotoxic effect (IC_50_, μg/mL)
FE	60.10 ± 0.00	0.005 ± 0.000	2.17 ± 0.15	0.007 ± 0.000	1790 ± 20	1200 ± 120	>400
LE	60.06 ± 0.00	0.006 ± 0.000	2.19 ± 0.14	0.007 ± 0.000	1102 ± 50	690 ± 10	>324.38
FPE	>3000	0.021 ± 0.000	1.28 ± 0.02	0.002 ± 0.000	NE	2610 ± 40	>400
FSE	160.10 ± 0.00	0.020 ± 0.000	1.53 ± 0.12	0.001 ± 0.000	1408 ± 0.00	1301 ± 10	>400
standard	3.00 ± 0.00^c^		2.43 ± 0.14^c^		10 ± 0.00^d^	14 ± 6^e^	

a IC_50_ values are half maximal inhibitory concentrations;

b ORAC values are determined for essential oil and extracts at 0.1 mg/mL; TE: Trolox equivalent;

c Standard used for DPPH assay: gallic acid,

d Standard used for β-carotene bleaching test: butylated hydroxyanisole,

e Standard used for α-amylase inhibition assay: acarbose. IC_50_ values higher than the maximum tested concentration was expressed as “>” values. NE: No effective inhibition was observed at the maximum tested concentration.

f FE: Flowers 70% (v/v) aqueous ethanol extract; LE: Leaf 70% (v/v) aqueous ethanol extract; FPE: Fruit pulp 70% (v/v) Aqueous Ethanol Extract; FSE: Fruit seeds 70% (v/v) aqueous ethanol extract.

Our study demonstrated that the flowers and the leaf extracts of *M. aquifolium* exhibited the strongest DPPH radical scavenging activity, with an IC_50_ value of 60.0 ± 0.00 μg/mL. Similarly, both leaves and flowers showed the highest ORAC activity reaching 0.007 ± 0.000 μmol TE/g_extract_. The fruit pulp extract yielded the highest TEAC value, measured as 0.021 ± 0.000 mM. Regarding β-carotene bleaching assay, the leaf extract exhibited the greatest efficacy, with an IC_50_ value of 1102 ± 50 μg/mL. In the CUPRAC assay, again the leaf and flower extracts demonstrated the highest reducing power, with values of 2.19 ± 0.14 and 2.17 ± 0.15 mM, respectively. Furthermore, α-amylase inhibition assays indicated that the leaf extract possessed the strongest inhibitory activity, with an IC_50_ of 690 ± 10 μg/mL. A significant positive correlation was observed between total phenolic content and antioxidant activity across all tested extracts.

According to earlier reported findings, the DPPH radical scavenging activity of *M. aquifolium* fruits extracted with 80% methanol reached 2.96 ± 0.12 mmol TE/100 g_fresh material_, indicating the antioxidant potential of the polar constituents.^80^ *Mahonia* berries’ phenolic and anthocyanin concentration mostly explains their antioxidant action; anthocyanins usually contribute the most. Phenolic and anthocyanin chemicals in *M. aquifolium* constitute more than 83% of the overall antioxidant action.^3,11,14,68,81,85–87^

These results corroborate the elevated phenolic content and antioxidant capacity of the *M. aquifolium* 70% (v/v) aqueous ethanol extracts, aligning with the significant biological activities noted in our present investigation.

The cytotoxic effects of the *M. aquifolium* extracts on NIH/3T3 cells were assessed through an MTT assay. According to the results at 400, 100, 10, and 1 μg.mL^–1^ concentrations of FE, FPE, FSE, and LE, NIH/3T3 cell viability altered by the following percentages at 24 h:, 75.36, 98.37, 105.51, and 114.60%; 77.78, 87.63, 89.45, and 97.38%; 57,83, 92.19, 105.14, and 107.50%, and 62.14, 86.87, 92.23, and 98.66%, respectively. At 48 h, the corresponding percentages were: 58.99, 97.33, 106.83, and 119.19%; 77.04, 91.91, 95.28, and 95.45%; 56.95, 100.99, 109.09, and 111.34%, respectively (Figure S1). Based on the obtained MTT assay data, FE, FPE, and FSE did not reach 50% inhibitory concentration within the tested range (IC_50_ > 400 μg/mL), indicating negligible cytotoxicity toward NIH/3T3 fibroblast cells. In contrast, LE exhibited a moderate, time-dependent cytotoxic effect, with an IC_50_ value of 324.38 μg.mL^–1^ after 48 h of treatment. The cytotoxic effects of the extracts on THP-1 cells were assessed at 400, 100, 10, and 1 μg mL^–1^ concentrations of FE, FPE, FSE, and LE. At 24 h, THP-1 cell viability changed by 104.82, 108.64, 112.32, and 112.92%; 89.66, 93.58, 96.79, and 99.31%; 84.57, 92.21, 109.30, and 114.41%; and 100.08, 105.38, 107.33, and 108.22%, respectively. At 48 h, the corresponding percentages were 82.98, 94.57, 105.84, and 109.48%; 86.03, 91.67, 94.18, and 98.68%; and 56.01, 85.08, 105.26, and 106.53%, respectively (Figure S2).

These results indicate that none of the extracts exhibited significant cytotoxicity toward THP-1 cells at the tested concentrations. In contrast, the slight increase in cell viability, particularly at lower concentrations, may suggest enhanced metabolic activity or cellular protection, which is consistent with the high antioxidant content of the extracts. Accordingly, all extracts demonstrated IC_50_ values greater than 400 μg mL^–1^, indicating negligible cytotoxic potential toward monocyte-derived cells. In the literature, *Mahonia* genus extracts generally exhibit low-to-moderate cytotoxicity when evaluated in fibroblast and THP-1 cells using the MTT assay.^15,19,88,89^ They are less toxic to normal cells compared to cancer cells and have antimetastatic and immunomodulatory potential. These findings support the selective cytotoxic and therapeutic potential of *Mahonia* extracts.

## 4 Conclusions

This study provides a comprehensive organ-specific phytochemical characterization of polar extracts obtained from the flowers, leaves, fruit pulp, and seeds of *M. aquifolium*. By combining UHPLC-HRMS/MS–based qualitative and quantitative analyses with complementary silylation-assisted GC-FID/MS profiling, distinct chemical patterns were revealed among different plant organs, highlighting marked differences in phenolic composition.

The applied analytical strategy enabled the reliable identification and quantification of major phenolic constituents, with chlorogenic acid emerging as a predominant compound across several organs. Notably, the separate investigation of fruit pulp and seeds represents a novel contribution, allowing the detection of compounds not previously reported for polar extracts of *M. aquifolium*, including the organ-specific occurrence of flavanomarein in seeds and the presence of 3-hydroxytyrosol in selected organs. These findings expand the current phytochemical knowledge of the species and underline the relevance of organ-resolved analytical approaches. Importantly, the pronounced organ-specific chemical differentiation observed in this study suggests that *M. aquifolium* is better suited for targeted, organ-based utilization rather than nonselective whole-plant exploitation. The distinct enrichment of specific phenolic compounds across flowers, leaves, fruit pulp, and seeds provides a rational basis for selecting individual plant organs according to the intended functional application. This organ-resolved perspective is particularly relevant for food, nutraceutical, and pharmaceutical sciences, as it may inform optimized processing strategies, formulation design, and functional product development.

The biological assays performed in this study were used as supportive tools to complement the chemical data. Antioxidant and cell-based assays were included as complementary evaluations and were interpreted cautiously in support of the chemical profiling data. Overall, the results emphasize the chemical diversity of *M. aquifolium* polar extracts and provide a solid analytical foundation for future investigations focusing on targeted bioactivity assessment, bioavailability, and safety evaluation.

## Supplementary Material



## Data Availability

Data used is available throughout the manuscript text and Supporting Information.

## Notes

Identified and quantified phenolic compounds in the 70% (v/v) aqueous ethanol extracts of *M. aquifolium* leaves, flowers, fruit pulps, and fruit seeds by UHPLC-HRMS/MS (Tables S1–S4) and cell viability assay results of the extracts on NIH/3T3 and THP-1 cell lines (Figures S1 and S2) (PDF)
