# Supplementary material for: Organ-Specific Metabolite Profiling of Mahonia aquifolium (Pursh) Nutt. Extracts by GC-FID/MS and UHPLC-HRMS/MS with Bioactivity Assessment
Source: ACS Omega. 2026 Mar 4;11(10):16558–70. doi: 10.1021/acsomega.5c12812 (PMC13000568; doi:10.1021/acsomega.5c12812)
Supplement: Supplementary file 1 [file ao5c12812_si_001.pdf]

Supporting Information

Organ-Specific Metabolite Profiling of *Mahonia aquifolium* (Pursh) Nutt. Extracts by GC-FID/MS and UHPLC-HRMS/MS with Bioactivity Assessment

Kübra Öğüt [a]*, Elif Kaya Tilki[b], Ana M. Troncoso[c], Temel Özek[a]

[a]Anadolu University, Department of Pharmacognosy, Faculty of Pharmacy, Eskişehir, 26470, Türkiye
[b]Anadolu University, Department of Pharmacology, Eskişehir, 26470, Türkiye
[c]Departamento de Nutrición y Bromatología, Toxicología y Medicina Legal. Facultad de Farmacia, Universidad de Sevilla, C/P García González No. 2, Sevilla 41012, Spain
*Corresponding author: kubraogut@anadolu.edu.tr (Kübra Öğüt)

Table S1. Identified and Quantified Phenolic Compounds in the 70% Ethanol Extract of *M. aquifolium* Leaves by UHPLC-HRMS/MS

Compound*	t_R (min)	Molecular formula	*m/z* (Expected)	*m/z* (Apex)	Mass error (ppm)	MS/MS fragments	$mg_{compound}/g_{dry\ extract}$ (medium ± std)
3-Hydroxytyrosol	2.64	$C_8H_{10}O_3$	153.05572	153.0556	-0.764	81.03464; 93.03469; 95.05029; 108.02177; 123.04531	1.20±0.11
4-Hydroxybenzoic acid	3.14	$C_7H_6O_3$	137.02442	137.02431	-0.823	65.03969; 93.03461; 137.02452	493.1±0.00
4-O-Caffeoylquinic acid	4.05	$C_{16}H_{18}O_9$	353.08781	353.08777	-0.117	93.03463; 135.04538; 173.04588; 179.03494; 191.0564	27.67±0.12
Caffeic acid	4.02	$C_9H_8O_4$	179.03498	179.03496	-0.124	89.0398;107.0503;134.03751;135.04536; 179.03522	20.65±0.25
Chlorogenic acid	3.83	$C_{16}H_{18}O_9$	353.08781	353.08771	0.290	59.0139; 85.02959; 93.03463; 127.04018; 191.0564	527.74±8.22
p-Coumaric acid	4.81	$C_9H_8O_3$	163.04007	163.04004	-0.190	65.03965; 91.05541; 93.03467; 119.05032	0.12±0.00
Protocatechuic acid	2.2	$C_7H_6O_4$	153.01933	153.01926	-0.480	65.00334;81.03461; 91.01897;108.0218; 109.02957	8.95±0.10
Apigenin-7-O-Glc	5.98	$C_{21}H_{20}O_{10}$	431.09837	431.09821	-0.381	63.02411;107.01395; 117.0347; 211.04031; 268.03818	11.00±0.80
Diosmetin	7.37	$C_{16}H_{12}O_6$	299.05611	299.05588	-0.777	63.02412; 65.00336; 151.00378; 284.03293	3.13±0.19
Isorhamnetin	7.34	$C_{16}H_{12}O_7$	315.05103	315.0513	0.857	107.01395; 243.03014;255.02951; 271.02521; 300.02777	0.10±0.00
Luteolin	6.85	$C_{15}H_{10}O_6$	285.04046	285.04047	-0.299	65.00336; 107.01401; 133.02963; 151.00374; 175.0401	38.78±0.74
Naringin	5.84	$C_{27}H_{32}O_{14}$	579.17193	579.17163	-0.515	579.1720; 459.1085; 271.0603; 151.0038; 119.0497; 177.0185	0.19±0.00

t_R (min) refers to the chromatographic retention time. Exact mass $(M-H)^-$ corresponds to the calculated monoisotopic mass of the deprotonated molecule. *m/z* (Expected) represents the theoretical mass-to-charge ratio, *m/z* (Apex) the experimentally observed peak, and Mass error (ppm) represents the mass difference between expected and observed *m/z* values, expressed in parts per million * Analytes were confirmed by comparison with pure standards. Standard deviations reported as 0.00 indicate values below the reporting precision (two decimal places).

Table S2. Identified and Quantified Phenolic Compounds in the 70% Ethanol Extract of *M. aquifolium* Flowers by UHPLC-HRMS/MS

Compound*	t_R (min)	Molecular formula	*m/z* (Expected)	*m/z* (Apex)	Mass error (ppm)	MS/MS fragments	$mg_{compound}/g_{dry\ extract}$ (medium ± std)
3-Hydroxytyrosol	2.64	$C_8H_{10}O_3$	153.05572	153.05568	-0.266	81.03464; 93.03469; 95.05029; 108.02177; 123.04531	19.15±0.53
4-Hydroxybenzoic acid	3.32	$C_7H_6O_3$	137.02442	137.02434	-0.600	65.03979; 93.03469; 137.0246	1.88±0.01
4-O-Caffeoylquinic acid	4.02	$C_{16}H_{18}O_9$	353.08781	353.08792	0.315	93.03463; 135.04535; 173.04573; 179.03519; 191.0564	92.28±2.06
Caffeic acid	4.05	$C_9H_8O_4$	179.03498	179.03497	-0.038	89.03982; 107.05034; 134.03751; 135.04533; 179.03522	12.35±0.27
Chlorogenic acid	3.83	$C_{16}H_{18}O_9$	353.08781	353.08786	0.142	59.0139; 85.02959; 93.03463; 127.0402; 191.0564	786.06±5.05
p-Coumaric acid	4.8	$C_9H_8O_3$	163.04007	163.04008	0.091	65.03979; 91.05544; 93.03466; 104.02676; 119.05033	1.22±0.05
Protocatechuic acid	2.2	$C_7H_6O_4$	153.01933	153.01929	-0.280	65.00311; 81.03467; 91.01907; 108.02183; 109.02959	18.81±0.12
Diosmetin	7.37	$C_{16}H_{12}O_6$	299.05611	299.05591	-0.674	107.014; 151.00385; 284.0329	0.58±0.01
Isorhamnetin	7.34	$C_{16}H_{12}O_7$	315.05103	315.0513	-0.208	243.02974; 255.02866; 271.02484; 300.02777	3.63±0.04
Luteolin	6.85	$C_{15}H_{10}O_6$	285.04046	285.04037	-0.299	65.00336; 107.01401; 133.02963; 151.00375; 175.04013	3.99±0.07
Vanillin	4.48	$C_8H_8O_3$	151.04007	151.04002	-0.306	92.02692; 108.02183; 136.01672; 151.04018	3.09±0.00

t_R (min) refers to the chromatographic retention time. Exact mass $(M-H)^-$ corresponds to the calculated monoisotopic mass of the deprotonated molecule. *m/z* (Expected) represents the theoretical mass-to-charge ratio, *m/z* (Apex) the experimentally observed peak, and Mass error (ppm) represents the mass difference between expected and observed *m/z* values, expressed in parts per million. * Analytes were confirmed by comparison with pure standards. Standard deviations reported as 0.00 indicate values below the reporting precision (two decimal places).

Table S3. Identified and Quantified Phenolic Compounds in the 70% Ethanol Extract of *M. aquifolium* Fruit Pulps by UHPLC-HRMS/MS

**Compound***	**t_R (min)**	**Molecular formula**	***m/z* (Expected)**	***m/z* (Apex)**	**Mass error (ppm)**	**MS/MS fragments**	**$mg_{compound}/g_{dry\ extract}$ (medium ± std)**
Caffeic acid	4.05	$C_9H_8O_4$	179.0350	179.0349	-0.464	89.03958; 107.0506; 134.03755; 135.04539; 179.03529	0.18±0.01
Chlorogenic acid	3.87	$C_{16}H_{18}O_9$	353.0878	353.0876	-0.636	59.01392; 85.02961; 93.03468; 127.04026; 191.05646	13.32±0.01
Protocatechuic acid	2.21	$C_7H_6O_4$	153.0193	153.0192	-0.879	65.00336; 81.03469; 91.01903; 108.02183; 109.02959	0.27±0.00
Catechin	3.59	$C_{15}H_{14}O_6$	289.0718	289.0718	0.166	109.0262; 123.04527; 137.02461; 203.07208; 245.08223	1.11±0.06
Luteolin	6.86	$C_{15}H_{10}O_6$	285.0405	285.0404	-0.192	65.00335; 107.01399; 133.02965; 151.00371; 175.04025	0.76±0.04

t_R (min) refers to the chromatographic retention time. Exact mass $(M-H)^-$ corresponds to the calculated monoisotopic mass of the deprotonated molecule. *m/z* (Expected) represents the theoretical mass-to-charge ratio, *m/z* (Apex) the experimentally observed peak, and Mass error (ppm) represents the mass difference between expected and observed *m/z* values, expressed in parts per million. * Analytes were confirmed by comparison with pure standards. Standard deviations reported as 0.00 indicate values below the reporting precision (two decimal places).

Table S4. Identified and Quantified Phenolic Compounds in 70% Ethanol Extract of *M. aquifolium* Fruit Seeds by UHPLC-HRMS/MS

Compound*	t_R (min)	Molecular formula	*m/z* (Expected)	*m/z* (Apex)	Mass error (ppm)	MS/MS fragments	$mg_{compound}/g_{dry\ extract}$ (mean ± std)
3-Hydroxytyrosol	2.64	$C_8H_{10}O_3$	153.05572	153.05563	-0.565	81.03471; 93.03469; 95.0535; 108.02186; 123.04533	4.64±0.01
4-Hydroxybenzoic acid	3.14	$C_7H_6O_3$	137.02442	137.02432	-0.711	65.03947; 93.0347; 137.02458	0.45±0.01
Caffeic acid	4.03	$C_9H_8O_4$	179.03498	179.03497	-0.294	89.0398; 107.05035; 134.03755; 135.04538; 179.03529	3.73±0.03
Chlorogenic acid	3.86	$C_{16}H_{18}O_9$	353.08781	353.08777	-0.117	59.01395; 85.02962; 93.03468; 127.04024; 191.05646	121.17±3.09
p-Coumaric acid	4.81	$C_9H_8O_3$	163.04007	163.04002	-0.283	91.05542; 93.03468; 119.05035	0.17±0.01
Protocatechuic acid	2.21	$C_7H_6O_4$	153.01933	153.01926	-0.480	65.00344; 81.0347; 91.01903; 108.02185; 109.0296	7.65±0.18
Apigenin-7-O-Glc	5.99	$C_{21}H_{20}O_{10}$	431.0984	431.09824	-0.381	63.02415; 107.01398; 117.03474; 211.04028; 268.03806	1.38±0.02
Catechin	3.59	$C_{15}H_{14}O_6$	289.07176	289.07184	0.271	109.02963; 123.04536; 137.02449; 203.07201; 245.08224	47.80±0.23
Diosmetin	7.38	$C_{16}H_{12}O_6$	299.05611	299.05588	-0.777	63.0415; 65.00338; 107.01399; 151.00372; 284.03302	0.89±0.01
Flavanomarein	4.41	$C_{21}H_{22}O_{11}$	449.10893	449.10883	-0.368	107.01401; 135.04526; 151.00383; 287.05655	46.18±0.57
Isorhamnetin	7.36	$C_{16}H_{12}O_7$	315.05103	315.05093	-0.305	107.01387; 243.0302; 255.02954; 271.0251; 300.02777	0.12±0.01
Luteolin	6.86	$C_{15}H_{10}O_6$	285.04046	285.04045	-0.129	65.00335; 107.01399; 133.02966; 151.003785; 175.03998	12.05±0.14

t_R (min) refers to the chromatographic retention time. *m/z* (Expected) represents the theoretical mass-to-charge ratio, *m/z* (Apex) the experimentally observed peak, and Mass error (ppm) represents the mass difference between expected and observed *m/z* values, expressed in parts per million. * Analytes were confirmed by comparison with pure standards. Standard deviations reported as 0.00 indicate values below the reporting precision (two decimal places).

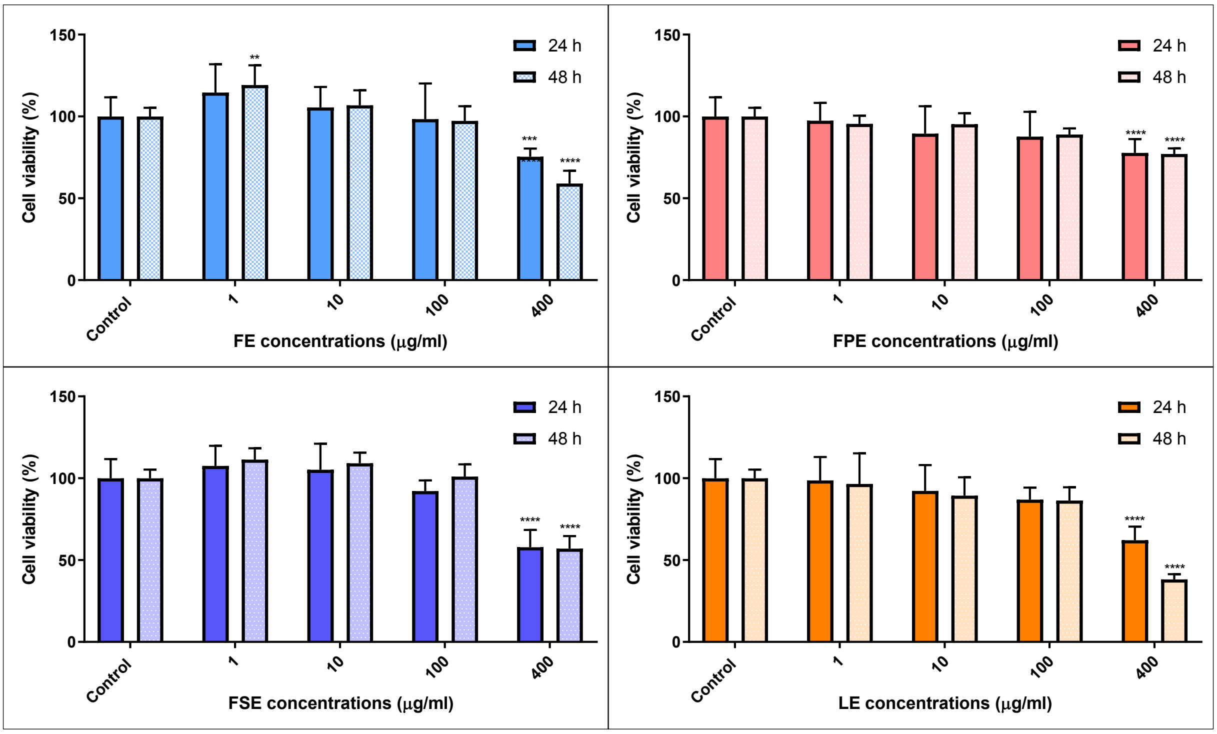

Figure S1. Cell viability assay results for varying concentrations (1, 10, 100, and 400 µg/mL) of FE, LE, FPE, and FSE on NIH/3T3 cells. Cell viability was evaluated after 24 and 48 h of treatment. Data are expressed as the mean ± SD from two independent experiments (n = 5 per treatment group). Significant differences are indicated as **$p < 0.01$, ***$p < 0.001$, and ****$p < 0.0001$, while non-significant differences are denoted as ns ($p > 0.05$). FE: Flowers 70% (v/v) aqueous ethanol extract; LE: Leaf 70% (v/v) aqueous ethanol extract; FPE: Fruit pulp 70% (v/v) aqueous ethanol extract; FSE: Fruit seeds 70% (v/v) aqueous ethanol extract.

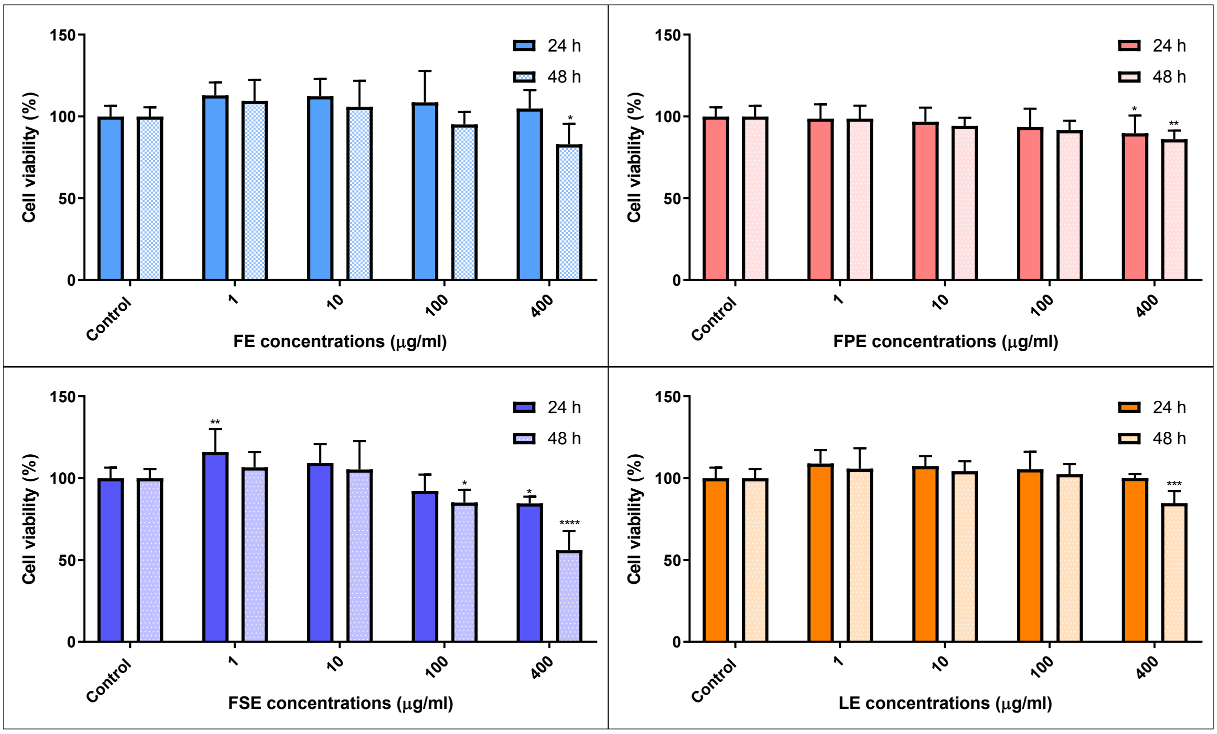

Figure S2. Cell viability assay results for varying concentrations (1, 10, 100, and 400 μg/mL) of FE, LE, FPE, and FSE on THP-1 cells. Cell viability was evaluated after 24 and 48 h of treatment. Data are expressed as the mean ± SD from two independent experiments (n = 5 per treatment group). Significant differences are indicated as $^{*}p < 0.05$, $^{**}p < 0.01$, $^{***}p < 0.001$, and $^{****}p < 0.0001$, while non-significant differences are denoted as ns ($p > 0.05$). FE: Flowers 70% (v/v) aqueous ethanol extract; LE: Leaf 70% (v/v) aqueous ethanol extract; FPE: Fruit pulp 70% (v/v) aqueous ethanol extract; fse: Fruit seeds 70% (v/v) aqueous ethanol extract.
